# PKC and PKA Regulate AChR Dynamics at the Neuromuscular Junction of Living Mice

**DOI:** 10.1371/journal.pone.0081311

**Published:** 2013-11-15

**Authors:** Isabel Martinez-Pena y Valenzuela, Marcelo Pires-Oliveira, Mohammed Akaaboune

**Affiliations:** 1 Department of Molecular, Cellular and Developmental Biology, University of Michigan, Ann Arbor, Michigan, United States of America; 2 Program in Neuroscience, University of Michigan, Ann Arbor, Michigan, United States of America; Georgia Regents University, United States of America

## Abstract

The steady state of the acetylcholine receptor (AChR) density at the neuromuscular junction (NMJ) is critical for efficient and reliable synaptic transmission. However, little is known about signaling molecules involved in regulating the equilibrium between the removal and insertion of AChRs that establishes a stable postsynaptic receptor density over time. In this work, we tested the effect of activities of two serine/threonine kinases, PKC and PKA, on the removal rate of AChRs from and the re-insertion rate of internalized recycled AChRs into synaptic sites of innervated and denervated NMJs of living mice. Using an in vivo time-lapse imaging approach and various pharmacological agents, we showed that PKC and PKA activities have antagonistic effects on the removal and recycling of AChRs. Inhibition of PKC activity or activation of PKA largely prevents the removal of pre-existing AChRs and promotes the recycling of internalized AChRs into the postsynaptic membrane. In contrast, stimulation of PKC or inactivation of PKA significantly accelerates the removal of postsynaptic AChRs and depresses AChR recycling. These results indicate that a balance between PKA and PKC activities may be critical for the maintenance of the postsynaptic receptor density.

## Introduction

The maintenance of a high density of nicotinic acetylcholine receptors (AChRs) at the postsynaptic membrane of a neuromuscular junction (NMJ) is essential for the effectiveness of synaptic impulse transmission. This high concentration of AChRs is established by rates of removal, re-insertion of recycled, insertion of newly synthesized and lateral diffusion of AChRs [1–3]. Several mechanisms have been implicated in the regulation of these rates, including synaptic activity, neural factors and receptor-associated scaffold proteins [1,2,4–9]. Several studies have also reported that serine/threonine kinases PKC and PKA activities are implicated in the clustering and stability of AChRs in cultured muscle [10–15]. However, it remains unknown at which steps of AChR trafficking PKC and PKA are involved.

PKA and PKC have been extensively studied in many cell types, including muscle cells. Predominantly, two isoforms of PKC are found to be expressed in skeletal muscle cells: conventional (c)PKCα [16], mainly localized in the cytosol and sarcolemma, and novel (n)PKCθ, mostly localized postsynaptically at the NMJ [17–20]. The skeletal muscle also abundantly expresses cAMP-dependent PKA, whose Rα-isoform is enriched in the NMJ region [21].

In the present work, we explored the role of the serine/threonine kinases PKC and PKA on AChR dynamics in living mice, particularly on the removal of AChRs from and the re-insertion of recycled AChRs into the postsynaptic membrane. We found that PKC and PKA have antagonistic effects on the removal of pre-existing receptors and the recycling of AChRs into the postsynaptic membrane. These results suggest that a tight balance between PKC and PKA activities is crucial for the stability of the postsynaptic receptor density.

## Results

### Effect of PKC on stability of AChR pools at the NMJ *in vivo*

**Figure 1:**
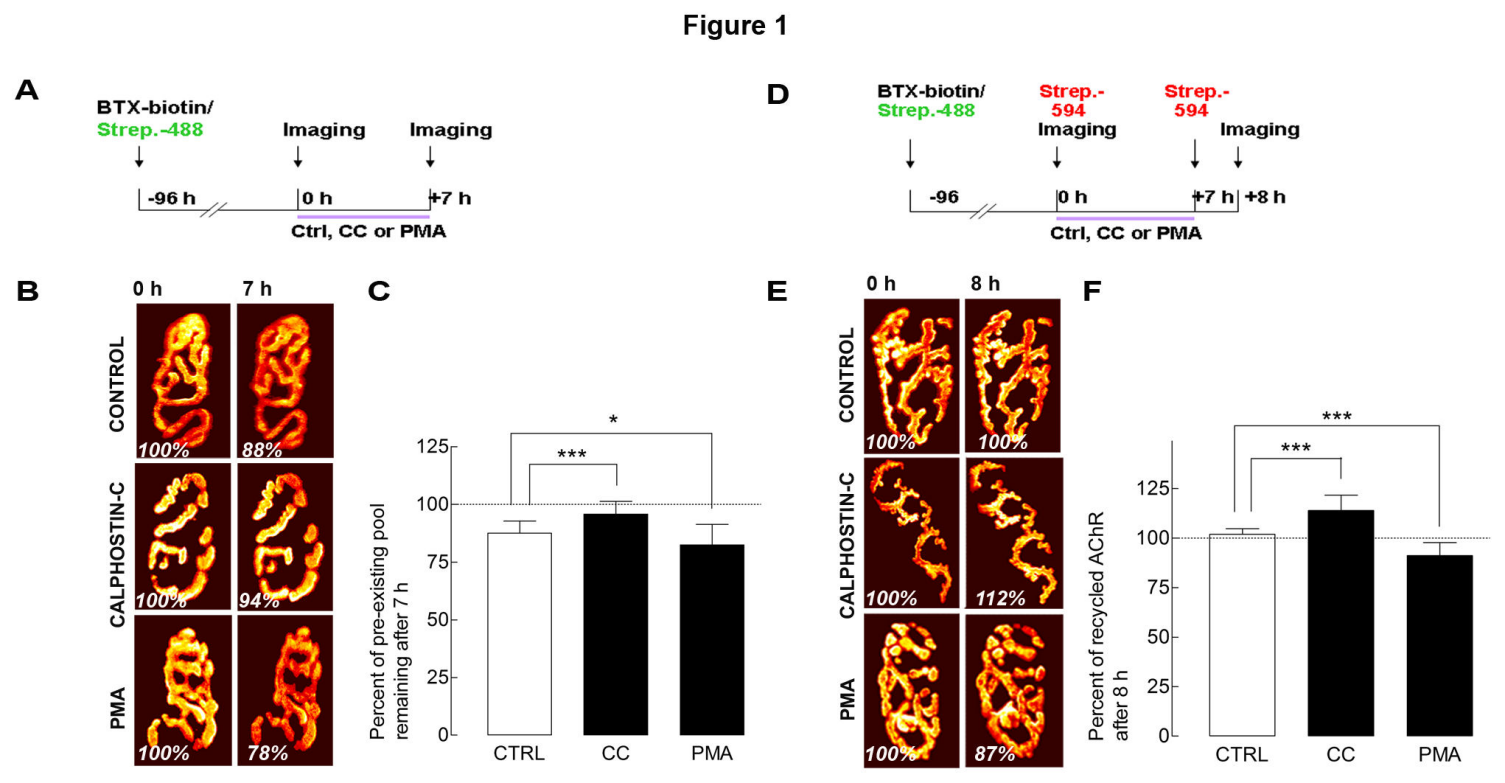
PKC activation accelerates the removal of receptors from synaptic sites *in* *vivo*. *A*, Labeling protocol for assessing the removal of preexisting AChRs from the postsynaptic membrane. Sternomastoid muscles were labeled with biotinylated α-bungarotoxin (BTX-biotin)/Alexa Fluor 488-streptavidin (strept-Alexa488; green). Four days later, superficial synapses were then imaged (time 0) and the sternomastoid muscles were bathed with or without PKC pharmacological agents for 7 h. At the end of the experiment, the same synapses were then imaged. *B*, Examples of control, and neuromuscular junctions treated with PKC inhibitor calphostin C (CC) and PKC activator phorbol-12-myristate-13-acetate (PMA), that were imaged at time 0 and 7 h later. The total fluorescence intensity of labeled preexisting AChRs was expressed as 100% at the time 0 and 7 hours later. Pseudo-color images provide a linear representation of the density of AChRs. Note that PKC inhibition with CC largely prevents the removal of preexisting AChRs while PKC activation accelerates their loss from postsynaptic membrane. *C*, Histogram summarizes the amount of preexisting receptors present at synaptic sites, obtained from many junctions by the approach shown in B. Each bar represents the mean percentage of original fluorescence intensity ± SD. *D*, Labeling method to analyze the insertion of recycled AChRs into the postsynaptic membrane. Sternomastoid muscles were labeled with BTX-biotin/strept-Alexa488; green. Four days later, muscles were bathed again with a saturating dose of strept-Alexa594, red, to selectively label the recycled receptors that had lost their initial strept-Alexa488 tag, while retaining BTX-biotin during the process of internalization and reinsertion. Superficial synapses were then imaged (time 0) and the sternomastoid muscles were bathed with PKC activators and inhibitors. At the end of the experiment, a second saturating dose of strept-Alexa594 was added to label receptors that have been recycled during the treatment. *E*, Example of control, and neuromuscular junctions treated with PKC inhibitor calphostin C (CC) and PKC activator PMA, that were imaged at time 0 and 8 h later. The total fluorescence intensity of labeled recycled AChRs was expressed as 100% at the time 0 and the fluorescence intensity 8 h later was compared with the fluorescence intensity of the synapse at the previous view. Note that PKC inhibition with CC increases the fluorescence intensity of recycled AChRs while PKC activation with PMA decreases their recycling. *F*, Histogram summarizes the amount of recycled receptors present at synaptic sites, obtained from many junctions by the approach shown in D. Each bar represents the mean percentage of original fluorescence intensity ± SD.*, p < 0.05; ***, p < 0.001.

Previous studies have reported that PKC is involved in the stability of AChRs [11,12,14,22,23]. In this work, we wanted to know which steps of AChR trafficking are regulated by PKC activity at the mature NMJs of living mice. To address this, we first tested whether activation or inhibition of PKC has any effect on the removal of AChRs from postsynaptic sites. To examine this, AChRs on the sternomastoid muscle were labeled with a non-saturating dose of biotinylated α‑bungarotoxin (BTX-biotin) followed by (green) streptavidin-Alexa Fluor4888 (strept-Alexa488) to saturate all biotin sites (see Methods). Four days later, the sternomastoid muscle was exposed and superficial synapses were imaged (time 0) (Figure 1A). The sternomastoid was bathed with PKC inhibitor, calphostin C, continuously for 7 hours and the same synapses were then re-imaged. The loss of fluorescence intensity from NMJs was assayed and compared with untreated synapses. In muscles treated with calphostin C, fluorescence intensity of pre-existing AChRs (not yet internalized) decreased by only 4% (96 ± 6% of original fluorescence; n = 33 NMJs, 3 mice), compared to untreated muscles (p < 0.001), where the fluorescence intensity decreased by 12% (88 ± 5% of original fluorescence; n = 19 NMJs, 3 mice) (Figure 1 B, C). In contrast, in muscles treated with PKC activator, phorbol-12-myristate-13-acetate (PMA), a widely used PKC activator [19,23], pre-existing AChRs fluorescence decreased significantly to 82 ± 9% (n = 39 NMJs, 5 mice), compared to untreated muscles (p < 0.05). (Figure 1 B, C).

Next, we asked whether PKC also affects the normal rate of recycling of previously internalized AChRs into the postsynaptic membrane. To this end, AChRs on the sternomastoid muscle were sequentially labeled with BTX-biotin, followed by a saturating dose of strept-Alexa488, as described in our previous work [3,24]. Four days later, recycled receptors were specifically labeled by adding (red) streptavidin-Alexa594 (strept-Alexa594) to the sternomastoid muscle (strept-Alexa594 binds to receptors that have lost their initial strept-Alexa488 tag while retaining BTX-biotin) [24]. Superficial synapses were imaged immediately (time 0), and the sternomastoid muscle was then bathed with calphostin C, a highly specific PKC blocker, to inhibit PKC [25,26] for the duration of the experiment (7 hours). At the end of the experiment, a second dose of strept-Alexa594 was added to label recycled receptors that had been inserted during the treatment of muscles and the same synapses were imaged for a second time (Figure 1 D). The fluorescence intensity of labeled recycled AChRs was measured before and after treatment. Quantification of recycled AChRs shows that after 7 hours of calphostin C treatment, the fluorescence intensity increased to 114 ± 8% (n = 57 NMJs, 7 mice) of their original fluorescence at time 0 (normalized at 100%) compared to untreated synapses where fluorescence remains unchanged, as previously described by Bruneau et al. [24] (102 ± 3%, n = 15 NMJs, p < 0.001, 3 mice) (Figure 1 E, F). As a second test of PKC inhibition, we used staurosporine (100 nM), a moderately potent PKC blocker, and found that the re-insertion of recycled AChRs at synaptic sites after 7 hours of treatment was also increased, albeit slightly less than with calphostin C (fluorescence intensity of recycled receptors was 106 ± 5% (n = 17 NMJs, 4 mice) versus untreated synapses, 99 ± 3% (n = 21 NMJs, 4 mice, p < 0.001).

The observation that PKC inhibition promotes the recycling of AChRs into synaptic sites prompted us to examine whether activation of PKC would depress the recycling of AChR. AChRs were labeled as described above, and four days later, the sternomastoid muscle was treated with PMA, and 7 hours after treatment, recycled receptors that had been inserted during the treatment of muscles were assessed. Quantification of fluorescently labeled recycled AChRs shows that the density of recycled receptors in muscles treated with PKC activator was significantly decreased (91 ± 7% of original fluorescence; n = 31 NMJs, 5 mice) when compared to untreated synapses (102 ± 3% of original fluorescence; n = 15 NMJs, 3 mice) (Figure 1 E, F).

**Figure 2:**
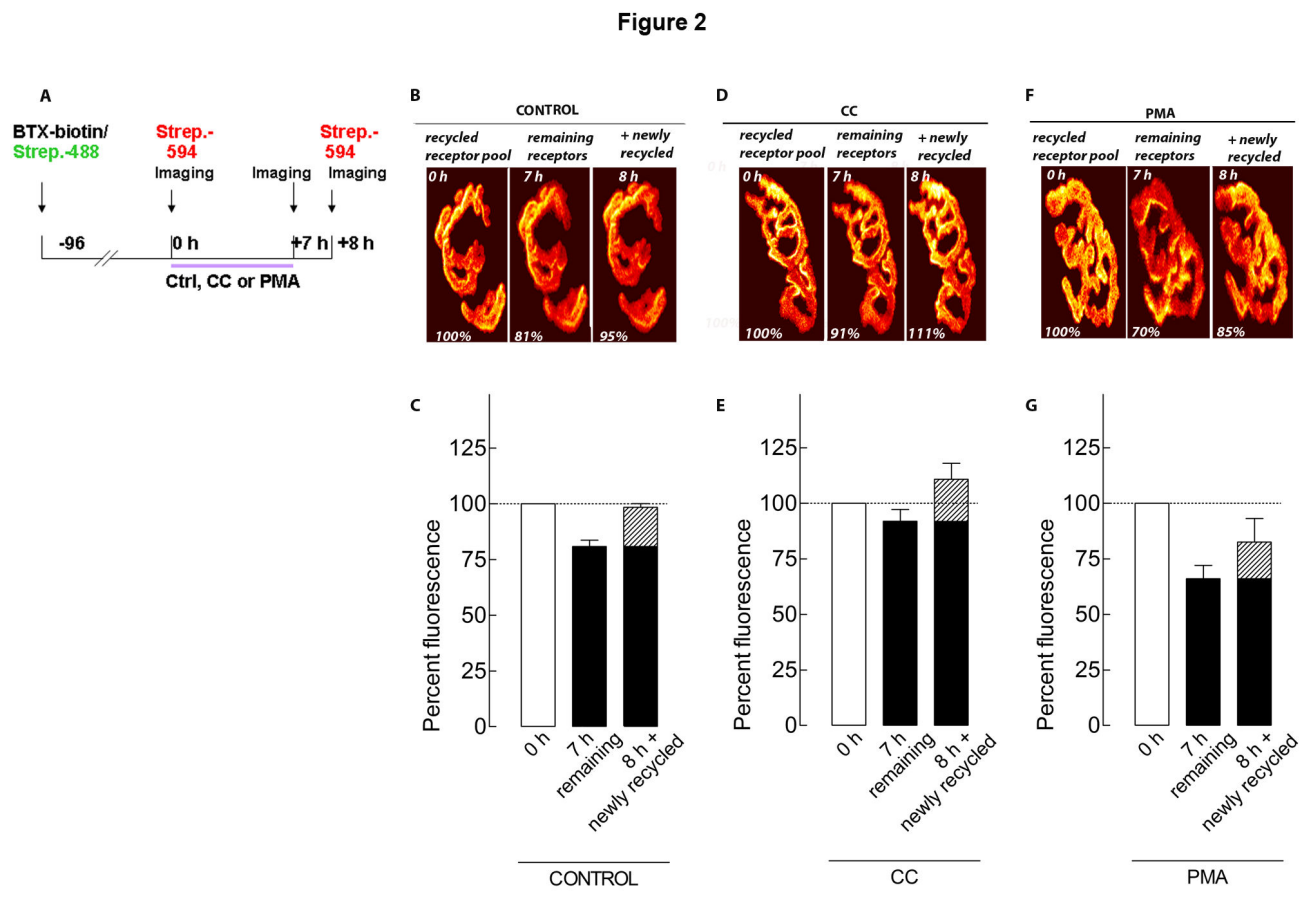
Activation of PKC accelerates the removal of recycled AChRs from the postsynaptic membrane *in* *vivo*. *A*, Labeling protocol of receptors as described above. Superficial synapses were imaged (time 0) and the sternomastoid muscles were treated with PKC inhibitors or activators for 7 h. At the end of the treatments, the same superficial synapses were imaged again to assess the loss of fluorescence from the first view (7 h). To test whether the loss of recycled matches the re-insertion of newly recycled receptors, the sternomastoid muscles were incubated with the same new fresh strept-Alexa594; (red) to selectively label the receptors that recycled during those 7 h. B, D, F, Examples of NMJs that were imaged immediately (time 0; recycled receptor pool), after 7 h of incubation with vehicle (***B***; control), calphostin C (***D***; CC), or phorbol-12-myristate-13-acetate (***F***; PMA), and then re-imaged after labeling of recycled receptors that were inserted during 7 h of treatment (newly recycled). *C*, *E*, *G*, Graphs summarizing data obtained from many synapses treated with vehicle (***C***; control), calphostin C (***E***; CC), phorbol ester (***G***; PMA) with the approach shown in B, D and F. Each bar represents the mean percentage of original fluorescence intensity ± SD.

Given the involvement of PKC activity on AChR recycling, we asked whether the increase of the recycled pool is due to an enhanced stability of recycled receptors in the membrane and/or to the promotion of the insertion of new recycled receptors. To distinguish between these two possibilities, AChRs on the sternomastoid muscles were labeled with BTX-biotin/strept-Alexa488 and four days later, the sternomastoid muscle was exposed and bathed with strept-Alexa594 to specifically label AChRs that had recycled after the initial labeling, and then the superficial synapses were imaged. The muscles were then treated with either PKC inhibitor calphostin C or PKC activator PMA for 7 hours; the same synapses were re-imaged, and their fluorescence intensity was measured. The muscles were bathed again with a second dose of strept-Alexa594, and the same synapses were imaged for a third time (Figure 2 A). Quantification of recycled AChR loss treated with calphostin C showed that the loss of fluorescence was largely prevented, as only 8% of labeled AChR was lost (92 ± 5% of original fluorescence; n = 9 NMJs, 3 mice) (Figure 2 D, E), compared to 19% in non-treated muscles (p < 0.05; 81 ± 3% of original fluorescence; n = 11 NMJs, 3 mice) (Figure 2 B, C). At the same synapses, the number of recycled receptors that had been inserted during the treatment was about 19% (111% - 92%) of the original fluorescence (up to 111 ± 7%; n = 9 NMJs, 3 mice) (Figure 2 D, E), similar (p > 0.05) to the 17% (98% - 81%) increase in control NMJs (up to 98 ± 2%; 11 NMJs, 3 mice) (Figure 2 B, C). In contrast, when muscles were treated with PKC activator PMA, the loss of recycled AChR was significantly increased (p < 0.001) to 34% of the original fluorescence (66 ± 6%; n = 8 NMJs, 3 mice) (Figure 2 F, G), compared to 19% in non-treated muscles. When the number of receptors recycled during the treatment was assessed, it was 17% (83% - 66%) of the original fluorescent pool (up to 83 ± 11%; n = 8 NMJs, 3 mice), not significantly different (p > 0.05) from the 17% (98% - 81%) in non-treated muscles. These results suggest that PKC regulates the AChR recycled pool by reducing the half-life of recycled receptors in the postsynaptic membrane.

### PKA activity antagonizes the effect of PKC on stability of AChR pools

**Figure 3:**
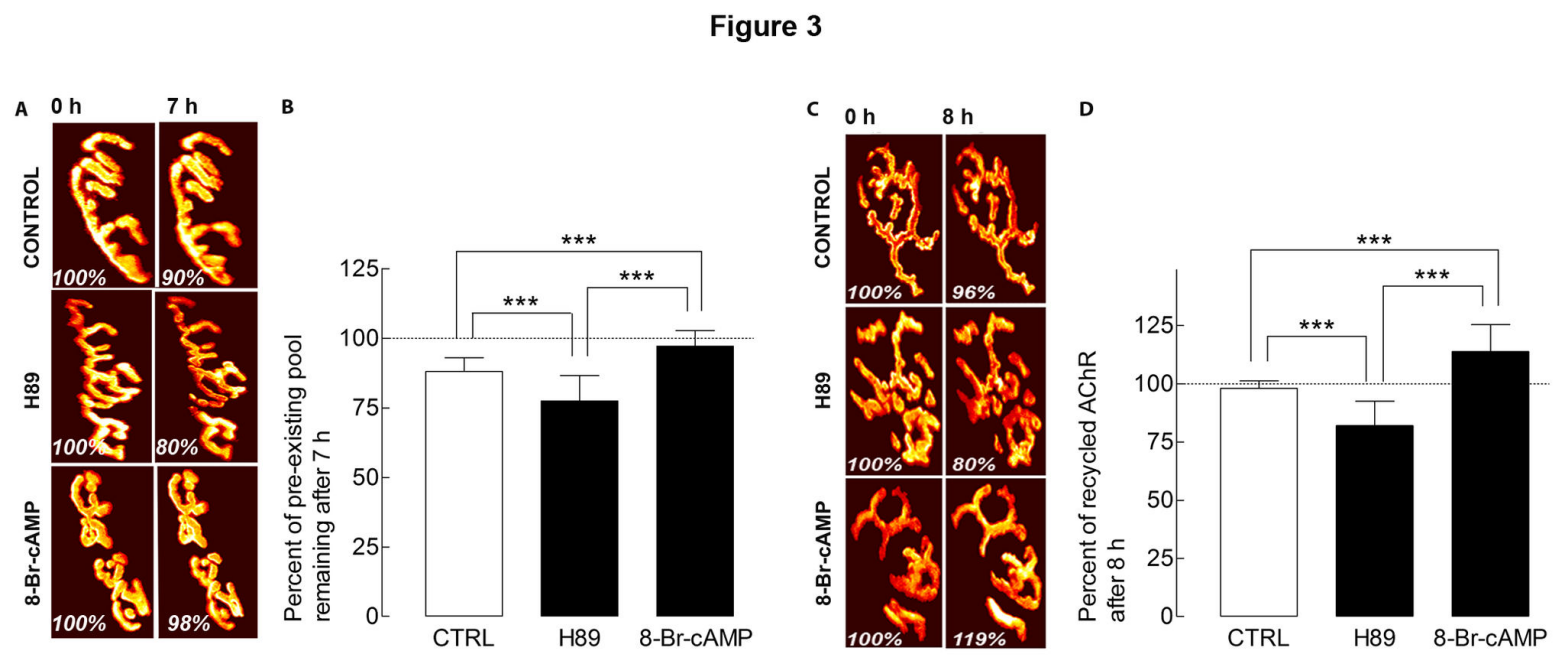
Stimulation of PKA increases the stability of AChRs at NMJ *in* *vivo*. Sternomastoid muscles were labeled as described above (Figure 1). *A*, Examples of two views of the same NMJ before and after treatment with PKA inhibitor and activator. Note that the loss of labeled preexisting receptors was largely prevented in muscles treated with PKA activator 8-Br-cAMP and was significantly accelerated in synapses treated with PKA inhibitor H89 compared to control synapses. *B*, Graph showing pre-existing receptors (retaining their strept-Alexa488 after initial labeling) from the same synapses as assessed in A. Each bar represents the mean percentage of original fluorescence intensity ± SD. ***, p < 0.001. C, Examples of recycled AChRs from control and NMJs incubated with PKA inhibitor H89 and PKA activator 8-Br-cAMP that were imaged at time 0 and 8 h later. D, Histogram summarizes the amount of recycled receptors present at synaptic sites, obtained from many junctions by the approach shown in A. Each bar represents the mean percentage of original fluorescence intensity ± SD. ***, p < 0.001.

PKA activity has been previously shown to enhance the stability of AChRs [12,27]; here, we sought to examine whether PKA activity has a similar effect on AChR dynamics at the NMJ of adult living mice. In particular, we investigated the effect of PKA on AChR removal and recycling. First, we examined the effect of PKA activity on the removal of pre-existing AChRs. AChRs on the sternomastoid muscle were labeled as described above and PKA activity was inhibited with the highly specific blocker H89 [28,29]. Quantification of the fluorescence intensity of pre-existing AChRs showed significant loss after 7 h (to 77 ± 9% of original fluorescence; n = 21 NMJs, 3 mice), compared to 88 ± 5% in non-treated muscles (n = 21 NMJs, 3 mice, p < 0.001). However, when PKA was stimulated with the metabolically stable activator of cAMP-dependent protein kinases, Sp-8-Br-cAMPS, receptor loss was minor (to 97 ± 7% of fluorescence remained; n = 23 NMJs, 3 mice, p < 0.001) (Figure 3 A, B).

Next, we evaluated whether PKA also affects AChR recycling. Quantification of recycled AChRs that had been inserted during treatment for 7 h with H89 showed that fluorescence decreased to 82 ± 11% (n = 59 NMJs, 6 mice) of the original value, compared to untreated synapses (98 ± 3%, n = 27 NMJs 4 mice, p < 0.001) (Figure 3 C, D). In contrast, when sternomastoid muscles were treated with Sp-8-Br-cAMPS, the fluorescence intensity of recycled receptors increased to 114 ± 12% (n = 55 NMJs, 6 mice) of their original fluorescence (p < 0.001 versus untreated synapses) (Figure 3 C, D).

**Figure 4:**
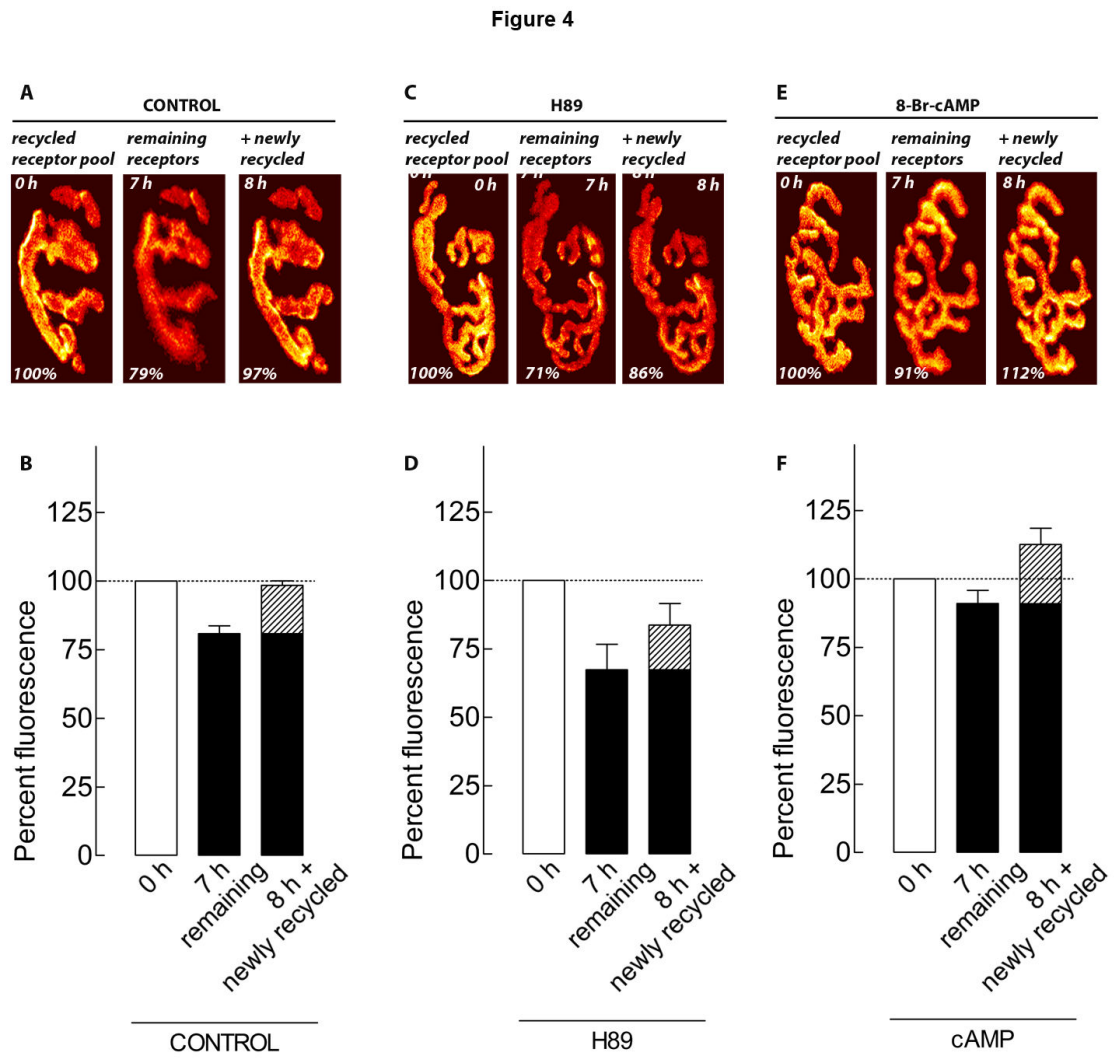
Activation of PKA prevents largely the removal of recycled AChRs at NMJ from postsynaptic membrane *in* *vivo*. Sternomastoid muscles were labeled with BTX-biotin/strept-Alexa488; green and 4 days later were bathed again with a saturating dose of strept-Alexa594; red as described above (Figure 2). The muscles were then treated with vehicle, PKA inhibitor (H89), PKA activator cAMP, for 7 h. the loss and insertion of recycled AChRs during treatment was assessed. *A*, *C*, *E*, Examples of NMJs that were imaged immediately (time 0; recycled receptor pool), after 7 h of incubation with vehicle (***A***; control), inhibitor (***C***; H89) or 8-Br-cAMP (***E***; cAMP) and then re-imaged after labeling of recycled receptors that were inserted during 7 h of treatment (newly recycled). *B*, *D*, *F*, Graphs summarizing data obtained from many synapses treated with vehicle (***B***; control), PKA inhibitor (***D***; H89), 8-Br-cAMP (***F***; cAMP) with the approach shown in A, C and E. Each bar represents the mean percentage of original fluorescence intensity ± SD.

To investigate whether PKA regulates the insertion of newly recycled receptors or their stability in synaptic sites, we measured the loss and insertion of recycled receptors during treatments with PKA inhibitors and activators. Inhibition of PKA with H89 accelerated the loss of recycled AChR, as 33% of the original fluorescence was lost (67 ± 9%; 10 NMJs, 3 mice), when compared (p < 0.01) to 19% in control synapses. The insertion of recycled receptors was 16% (84% - 67%) of the original labeled pool (up to 84 ± 8%; 10 NMJs, 3 mice) (Figure 4 C, D), which was not significantly lower than 17% (98% - 81%) in control muscles (p > 0.05) (Figure 4 A, B). When PKA was activated with Sp-8-Br-cAMPs, loss of recycled AChR was only 9% the of original fluorescence (91 ± 9%; 17 NMJs, 4 mice) (Figure 4 E, F), reduced from 19% in control NMJs (p < 0.001). Insertion of newly recycled receptors was 22% (113% - 91%) of the original labeled pool (up to 113 ± 6%; 17 NMJs, 4 mice), but not significantly higher than the 17% in control NMJs. 

### PKC and PKA regulate removal of AChRs from synaptic sites and AChR recycling through a similar pathway

**Figure 5:**
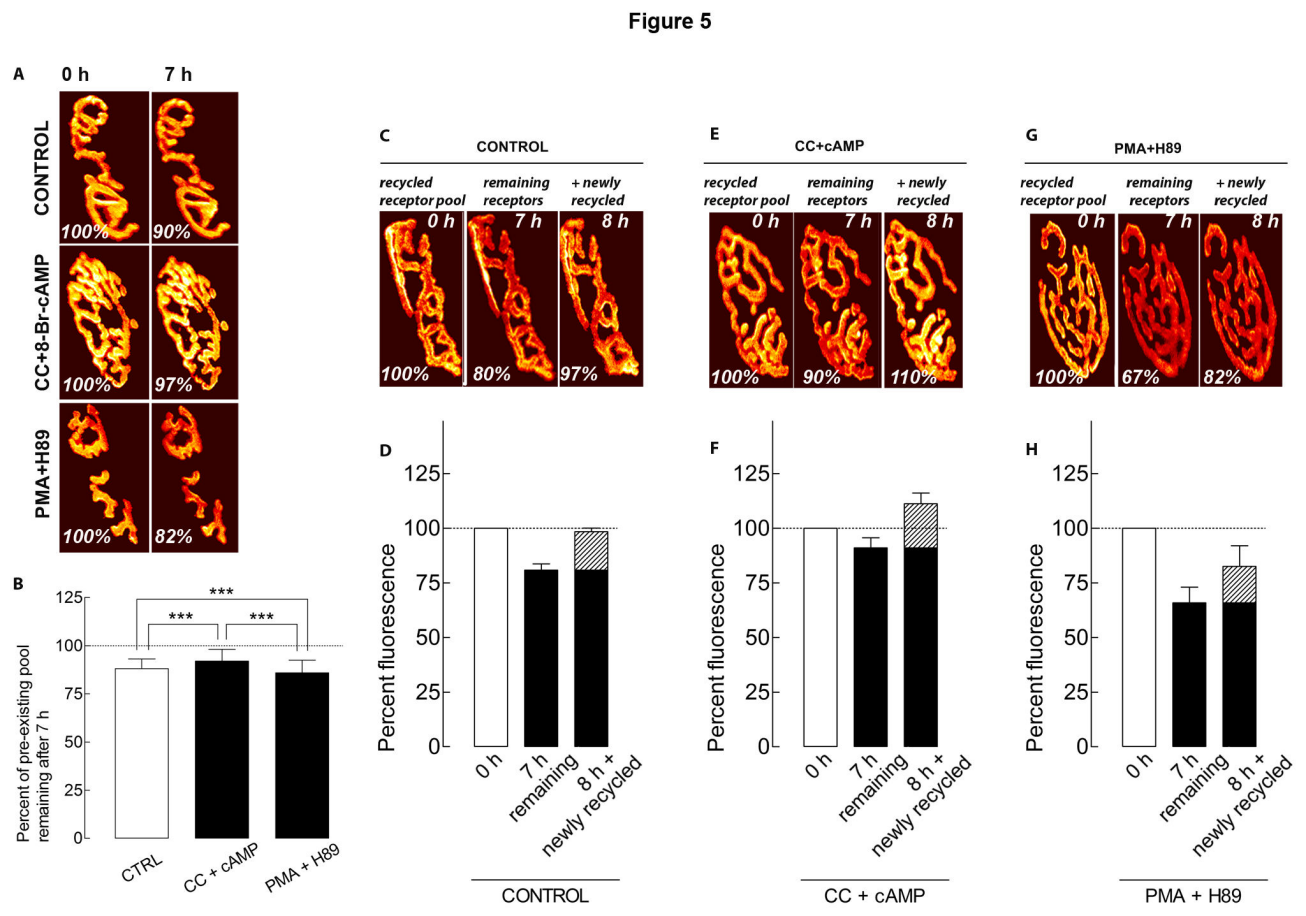
PKC inhibition and PKA activation do not act synergistically on the stability of receptors. *A*, Examples of two views of the same labeled pre-existing AChRs (AChRs that are not yet internalized) before (time 0) and after treatment (7 h later) with both PKC inhibitor CC and PKA activator 8-Br-cAMP, or PKC activator PMA and PKA inhibitor H89. Note that PKC inhibition in combination with PKA activation significantly decreased the removal of labeled preexisting AChRs compared to non-treated synapses but no more than one single treatment (Figures 1 and 3). Simultaneous PKC activation and PKA inhibition treatment accelerate the removal of preexisting AChRs, but comparable to one single treatment (Figures 1 and 3). *B*, Histogram summarizes the amount of preexisting receptors present at synaptic sites, obtained from many junctions by the approach shown in A. Each bar represents the mean percentage of original fluorescence intensity ± SD. ***, p < 0.001. *C*, *E*, *G*, Examples of NMJs (***C***; control, ***E***; CC + cAMP, ***G***; PMA+H89), showing that the loss of labeled recycled pool is also affected by PKC and PKA treatment. Note that the loss of recycled AChRs after 7 hours is prevented in the CC+cAMP treatment, but is increased in the PMA+H89 treatment, though the results are similar to each treatment alone (Figures 1 and 3). *D*, *F*, *H*, Graphs summarizing data obtained from many synapses with the approach shown in C, E and G. .

Next, we asked whether PKC and PKA activities have a synergistic effect on AChR removal and recycling. We first examined the effect of a PKC inhibitor and a PKA stimulator on the removal of AChRs from the same synapses. In muscles treated concomitantly with calphostin C and Sp-8-Br-cAMP, the fluorescently labeled pre-existing AChRs decreased by 8% (92 ± 6% of the original fluorescence; n = 15 NMJs, 4 mice) (Figure 5 A, B), similar (p > 0.05) to either treatment alone, suggesting no additive effects of the agents used on AChR removal. In the second set of experiments, muscles were treated with both the PKC activator PMA, and PKA inhibitor H89. When both were used in combination, the loss of AChRs was 14% (86 ± 7% of the original fluorescence; 8 NMJs, 3 mice) (Figure 5 A, B), which was not different (p > 0.05) from either treatment alone. We also investigated the combined effect of PKC and PKA on AChR recycling. Treatment with calphostin C and cAMP did not further reduce removal of recycled AChR (91 ± 5%, 13 NMJs, 3 mice) nor increase insertion (20%; 111% - 91%) (Figure 5 E, F) beyond any treatment alone (up to 111 ± 5%; 14 NMJs, 3 mice). Similarly, when both PMA and H89 were added together, removal of recycled AChR (68 ± 9%, 9 NMJs, 3 mice) or their insertion (16%, up to 84 ± 9%; 9 NMJs, 3 mice) (Figure 5 G, H) were affected similarly as when the treatments were isolated.

### Effect of PKC and PKA activities on AChR dynamics in denervated synapses

**Figure 6:**
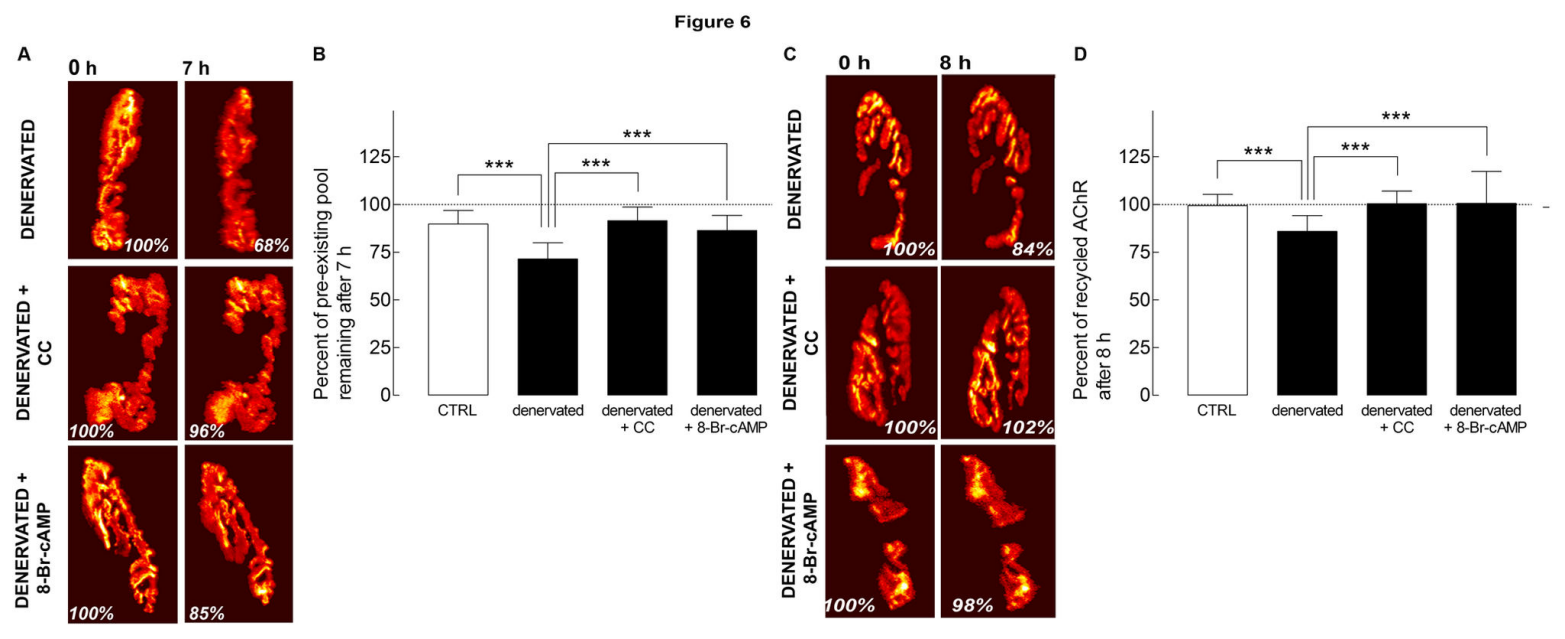
PKC inhibition and PKA activation restore recycled AChRs at denervated synapses. Denervated sternomastoid muscles (four days after denervation) were labeled with BTX-biotin/strept-Alexa488 and 3 days later, superficial synapses were then imaged (time 0) and the sternomastoid muscles were bathed with PKC inhibitor calphostin C or PKC activator phorbol-12-myristate-13-acetate (PMA) for 7 h. At the end of the experiment, the same synapses were then imaged. *A*, Example of denervated NMJs, non-treated or treated with CC and 8-Br-cAMP, imaged at time 0 and 7 h later. Fluorescence intensity of both treated denervated synapses increased compared to untreated denervated NMJs. *B*, Histogram summarizing the fluorescence measurements obtained from many NMJs by the approach shown in A. Each bar represents the mean percentage of original fluorescence intensity ± SD. ***, p < 0.001. C, Examples of recycled AChRs from the same denervated synapses as assessed by the change in fluorescence over the 7 h period of the experiments. Fluorescence intensity of labeled recycled receptors from denervated synapses was less than CC or 8-Br-cAMP treated denervated synapses. D, Graph showing the fluorescence measurements of recycled receptors obtained from many synapses by the approach shown in C. Each bar represents the mean percentage of original fluorescence intensity ± SD. ***, p < 0.001.

Previous studies have shown that in denervated muscles the loss of receptors is accelerated and only few internalized AChRs were able to recycle back into the synaptic original sites [3,9]. Here we asked whether PKC or PKA activity could prolong the metabolic stability of receptors in the postsynaptic membrane and promote the recycling of internalized ones. Receptors on denervated sternomastoid muscles (four days after nerve section) were labeled with BTX-biotin followed by strept-Alexa488 and three days later (seven days after denervation), the sternomastoid muscle was exposed and superficial synapses were imaged, and muscles were bathed with either PKC inhibitor calphostin C or PKA activator Sp-8-Br-cAMPS (both agents have been shown to largely prevent the removal of AChRs from innervated synapses, see Figures 1 and 3) for the duration of the experiment. Seven hours after treatment, the same synapses were re-imaged and changes in fluorescence intensities of labeled AChRs before and after treatment were assessed. In muscles treated with calphostin C, the loss of fluorescence intensity of pre-existing AChRs was only 8% of the original fluorescence (92 ± 7%, n = 18 NMJs, 3 mice, p < 0.001) compared to 29% of receptor loss in non-treated denervated synapses (71 ± 9%; n = 22 NMJs, 3 mice, p < 0.001). Similar results were obtained when denervated muscles were treated with PKA activator Sp-8-Br-cAMP, the loss was 14% of the original fluorescence; 86 ± 8%; n = 22 NMJs, 3 mice, p < 0.001 compared to non-treated denervated synapses) (Figure 6 A, B).

Given that PKC inhibition and PKA activation were able to promote AChR recycling, we asked whether these treatments could rescue AChRs from degradation and promote their recycling into denervated NMJs. To test this, denervated sternomastoid muscle (four days after nerve section) was labeled as described above and three days later, recycled AChRs that had been inserted after the initial labeling were imaged. In muscles treated with calphostin C, the fluorescence intensity of recycled receptors at the NMJs increased to 100 ± 7% (n = 30 NMJs, 3 mice) compared to untreated denervated synapses 86 ± 8% (n = 26 NMJs, 3 mice, p < 0.001) after 7 h. Similarly, treatment of muscles with PKA activator Sp-8-Br-cAMPS increased the number of recycled AChRs to 101 ± 17% of original fluorescence (n = 23 NMJs, 3 mice, p < 0.001 compared to non-treated denervated, 86 ± 8%) (Figure 6 C, D).

## Discussion

In this work, we show that the serine/threonine kinases PKA and PKC have antagonistic effects on the removal of pre-existing AChRs and the size of the recycled pool of AChRs at mature innervated and denervated neuromuscular junctions. Particularly, we show that inhibition of PKC or stimulation of PKA promotes the recycling of internalized AChR into synaptic sites and the anchoring of receptors at the postsynaptic membrane, while stimulation of PKC or inhibition of PKA depresses the recycling of AChR and accelerates the removal rate of receptors from the postsynaptic membrane. Furthermore, we show that inhibition of PKC and stimulation of PKA have no synergistic effects on AChR dynamics.

The present experiments show that both PKA and PKC kinase activities are linked to the trafficking and stability of AChRs. However, it is difficult to determine whether this process is a direct consequence of receptor phosphorylation or an indirect effect induced by phosphorylation of other proteins by PKA and PKC (effector molecules involved in internalization or proper delivery, for example). Previous studies have shown that all AChR subunits are subject to phosphorylation by different kinases. For instance, α and δ subunits are phosphorylated by PKC, δ and γ/ε are phosphorylated by PKA [30,31]. Thus, it is tempting to speculate that phosphorylation of AChR subunits can either promote or alter the trafficking and metabolic stability of AChR. For instance, when cultured myotubes were treated with PKC activators, receptor clusters failed to form in response to agrin, the insertion of new receptors in the membrane was impaired, and the disassembly of preexisting AChR clusters was enhanced [14,23,32,33]. Similarly, when PKC was overexpressed in muscle cells, the stability of receptors was reduced [34]. Conversely, inhibition of PKC activity (either by pharmacological agents or by genetic manipulations) enhanced the stability of receptor clusters in cultured myotubes and in living mice. Notably, in mice deficient in PKCθ isoform, the disassembly of receptor clusters (redistribution and dispersion), which normally occurs during the early stage of postnatal development, was delayed [22]. The phosphorylation of δ subunit in this mutant mouse is reduced [11], suggesting that the loss of PKC activity enhances the stability of receptors (at least through the phosphorylation state of δ subunit). It is possible that, in mature synapses, changes in the phosphorylation state of δ subunit by manipulations of PKC may have an effect on the fate of internalized AChRs (either degradation or recycling) and those anchored in the postsynaptic membrane (they remain stable or disassemble).

The current work shows that not only is PKC involved in receptor stability, but also PKA. Stimulation of PKA promotes both the recycling and stability of AChRs. Consistent with these observations, previous studies have reported that PKA stabilizes the receptors on the surface of cultured myotubes [12] and in cultured explants denervated diaphragms from mouse [27]. It is conceivable that phosphorylation of ε [15] or δ subunits (sites that are different from PKC phosphorylation sites) by PKA may stabilize the clustering of AChRs. It is also plausible that phosphorylation of scaffold proteins by PKA or other kinases may play a critical role in the stability of AChRs. Along these lines, it was reported that the loss of tyrosine phosphorylation of α-dystrobrevin reduces the stability of agrin-induced AChRs in cultured myotubes and in mice deficient in neuregulin receptors (*erb2/4* ^*-/-*^) [35]. Recently, it was suggested that PKA is also involved in the recycling of AChRs through its interaction with myosin Va, and in the stability of AChRs in the postsynaptic membrane through its anchoring by rapsyn [36,37]. It is also possible that phosphorylation of other effector molecules by PKA may play an important role in the sorting and proper delivery of AChRs to the plasma membrane. In the central nervous system, PKA activity has also been found to regulate AMPAR trafficking and insertion as its inhibition reduces AMPAR insertion and synaptic strength [38]. While it appears that the phosphorylation of receptors, receptor associated-scaffold proteins, and/or effector molecules by PKC and PKA are instrumental for the stability of AChRs, further studies are warranted to investigate when and how antagonistic effects of PKA and PKC are linked to receptor stability and trafficking. 

Finally, our findings suggest that PKA and PKC do not have synergistic effects on the removal of AChRs from or recycling into the postsynaptic membrane (Figure 5). This implies that these kinases might act to regulate receptor removal and recycling through a similar, overlapping pathway. In the present work, our quantitative fluorescence assay is not sensitive enough to test the effect of PKC and/or PKA on the synthesis of new AChRs over the short time window of our experiments. Since the detection of the pool of newly synthesized receptors requires that all pre-existing AChRs be completely saturated with α-bungarotoxin (and these synapses are then non-functional), insertion of new AChRs is heavily depressed [7].

Based on this and on previously published work, it appears that PKC and PKA act on a pathway distinct from CamKII, since when muscles were treated with KN93 (an inhibitor that blocks CamKII activity), PKC activator PMA and PKA inhibitor H89, the loss of AChRs from the synaptic membrane was increased significantly compared to PMA and H89 alone. While the mechanism by which these kinases activity control AChR removal and recycling is not known, it is possible that these kinases act on different receptor subunits and/or substrate proteins involved in anchoring and/or clustering receptors at synapses. Of note, all of these kinases are found to be concentrated at the postsynaptic membrane of the NMJ with different localizations; most notably, muscle specific CaMKII βm is precisely co-localized with receptors at the crests of the junctional folds [9]. Thus, it is conceivable that a spatial cellular compartmentalization of kinases in the postsynaptic density may play an important role in the trafficking and stability of receptors. Overall, the current work and other studies suggest that a balance between kinases (phosphorylation by PKC, PKA or CamKII) is important in controlling the molecular dynamics of AChRs at mature neuromuscular synapses.

## Methods

### Receptor pools labeling and neuromuscular junction imaging in living mice

This study was carried out according to the recommendations in the NIH Guide for the Care and Use of Laboratory Animals. The protocol was approved by the University Committee on the Use and Care of Animals of the University of Michigan (protocol number 3939). Non-Swiss Albino adult female mice (6–10 weeks old, 25–30 g) were anesthetized with an intraperitoneal injection of a mixture of 80 mg/kg ketamine and 20 mg/kg xylazine and the sternomastoid muscle was exposed, labeled, and the whole animal was placed on its back on the stage of a customized epifluorescence microscope as described previously [3,39–41]. Superficial neuromuscular junctions were imaged with a water-immersion objective (x20 UApo 0.8 NA Olympus BW51; Optical Analysis Corp.) 

The recycled receptor pool was identified using a method of labeling that allows one to selectively label recycled receptors, as described in our previous work in detail [3,24]. Briefly, receptors on the sternomastoid muscle were labeled with biotinylated bungarotoxin (BTX-biotin) (5 μg/ml, 30 min; Invitrogen) followed by a single saturating dose of streptavidin-Alexa Fluor 488 (strept-Alexa488; green; 10 μg/ml, 3 h; Invitrogen). A second color of (red) streptavidin Alexa-594 (10 μg/ml, 10–30 min) was then added to the sternomastoid muscle to be sure that all biotin sites are saturated. Four days later (after initial labeling, to allow more internalization of AChRs and formation of a sizeable pool), the mouse was anaesthetized and the recycled AChR pool on the sternomastoid muscle was specifically labeled with strept-Alexa 594 (10 µg/ml, 1 h) (receptors that had lost their streptavidin tag and were re-inserted in the synapses with their BTX-biotin tag). Superficial synapses were then imaged and re-imaged at the end of the experiment and their fluorescence intensities were measured. Experiments showing that the dissociation of streptavidin from biotin does not occur on the surface of the muscle cells but instead inside the muscle fiber were worked out in our previously published work [3,9,24]. 

### Pharmacological treatment

To test the effect of PKC on the removal of pre-existing AChRs form the postsynaptic membrane and the insertion of internalized recycled AChRs into synaptic sites, several experiments were performed. In the first series of experiments, the sternomastoid muscle was bathed with calphostin C (5 μM; Sigma), a potent, selective light-activated inhibitor for PKC isolated from the fungus *Cladosporium cladosporioides* [25,26,42]. Staurosporine (100 nM; Sigma), an agent that blocks a broad spectrum of kinases depending on the concentration was also used to block PKC. In a second series of experiments, we used phorbol-12-myristate-13-acetate (PMA), (200 nM; Sigma) [43], a pharmacological agent that stimulates PKC. 

Stimulation of PKA was performed by using the membrane-permeant and metabolically resistant agonist 8-bromoadenosine-3’-5’-cyclic monophos-phorothioate, Sp-8-Br-cAMP, (1 mM; BIOLOG) [44]. Inhibition of PKA activity was performed by using H89 (5 μM; Sigma) [45].

### Muscle denervation

Adult mice were anaesthetized, the sternomastoid was exposed and the nerve was excised by removing a 5 mm piece to prevent a possible re-innervation. Four days after denervation, the sternomastoid muscled was bathed with BTX-biotin followed by a saturating dose of streptavidin (strept-Alexa488). Three days after the initial labeling, the mouse was reanesthetized and the sternomastoid muscle was bathed with strept-Alexa594 (to label recycled nAChRs), and superficial synapses were imaged. PKC and PKA activators and inhibitors were used and the pre-existing receptor removal rate and recycled pool number were measured after 7 hours of drug treatments. 

### Quantitative fluorescence imaging

Quantitative fluorescence imaging was used to measure the fluorescence intensity of labeled receptor pools [7,9,39]. Briefly, images were calibrated to a non-fading reference standard to compensate for spatial and temporal changes in the light source and camera between imaging sessions at different time points. The same fluorescent ligands were repetitively imaged and as long as we verified that the image pixel intensity was not saturated, it was possible to get an accurate quantitative measurement of the relative number of nAChRs. Images were analyzed with algorithms for IPLAB (Scanalytics) and Matlab (The Mathworks). Background fluorescence was determined by manual selection of a boundary region around the each NMJ and subtracting it from the original image, and the mean of the total fluorescence intensity (which corresponds to receptor density) was measured [39].
